# Prevalence, Demographic, and Clinical Correlates of Comorbid Depressive Symptoms in Chinese Psychiatric Patients With Alcohol Dependence

**DOI:** 10.3389/fpsyt.2020.00499

**Published:** 2020-06-03

**Authors:** Hui Huang, Zhigan Zhu, Hongxian Chen, Kui Ning, Ruiling Zhang, Wei Sun, Bing Li, Haifeng Jiang, Wenzheng Wang, Jiang Du, Min Zhao, Zhihua Yi, Jing Li, Rongxin Zhu, Shuiping Lu, Shiping Xie, Xiaoping Wang, Wei Fu, Chengge Gao, Wei Hao

**Affiliations:** ^1^Affiliated Wuhan Mental Health Center, Tongji Medical College, Huazhong University of Science and Technology, Wuhan, China; ^2^Institute of Psychology, Chinese Academy of Sciences, Beijing, China; ^3^Mental Health Institute of the Second Xiangya Hospital, Central South University, The China National Clinical Research Center for Mental Health Disorders, National Technology Institute of Psychiatry, Key Laboratory of Psychiatry and Mental Health of Hunan Province, Changsha, China; ^4^Department of Psychiatry, Henan Mental Hospital , Xinxiang, China; ^5^Department of Psychiatry, Peking University Sixth Hospital, Beijing, China; ^6^Department of Psychiatry, Shanghai Mental Health Center, Shanghai, China; ^7^Department of Psychiatry, West China Hospital, Sichuan University, Chengdu, China; ^8^Department of Psychiatry, Nanjing Brain Hospital, Nanjing, China; ^9^Department of Psychiatry, Hubei General Hospital, Wuhan, China; ^10^Department of Psychiatry, The First Affiliated Hospital of Xian Jiaotong University, Xian, China

**Keywords:** alcohol dependence, depressive symptoms, prevalence, Beck Depression Inventory, Alcohol Use Disorders Identification Test

## Abstract

**Background:**

Depressive symptoms are common among psychiatric patients with alcohol dependence (AD). However, the prevalence and clinical correlates of comorbid depressive symptoms are less well studied in Chinese Han patients.

**Methods:**

In this hospital-based survey, we recruited 378 psychiatric patients diagnosed with AD according to the Diagnostic and Statistical Manual of Mental Disorders-Fourth Edition (DSM-IV). All patients completed the Beck Depression Inventory (BDI) to evaluate depressive symptoms and the Alcohol Use Disorders Identification Test (AUDIT) to assess the severity of drinking.

**Results:**

Compared to patients without depressive symptoms, 48.9% (185/378) of the patients with comorbid depressive symptoms were younger, had a more unstable marital status, had a higher AUDIT total score, and had a higher adverse consequences subscore (all *P* < 0.05). Further logistic regression analysis showed that unstable marital status (Odds ratios [OR] = 2.20, 95% confidence interval [CI] 1.21–3.99) and AUDIT total score (OR=1.07, 95% CI 1.03–1.11) were significantly associated with depressive symptoms.

**Conclusions:**

Our findings indicate high comorbidity between AD and depressive symptoms in Chinese psychiatric patients. Moreover, some variables are correlates of comorbid depressive symptoms. Particular attention should be paid to the early detection and intervention for this comorbid condition and its risk factors.

## Introduction

Alcohol dependence (AD) and depression commonly co-occur (1, 2). The prevalence of their co-occurrence in individuals with AD fluctuates markedly, according to how depressive symptoms are measured or defined. Epidemiologic data showed that the comorbidity rate in the general population varied between 15% and 28% (1, 3–5), whereas in clinical samples, this rate was much higher, ranging from 43% to 48% (6, 7). Moreover, AD is significantly associated with depression. For example, Lai et al. conducted a meta-analysis of 22 community-based studies and found that the pooled odds ratio for AD and major depression was 3.09 (95% confidence interval [CI] 2.38–4.03) (8). In another hospital-based survey, alcohol abuse was strongly associated with depressive symptoms (OR 2.58, 95% CI 1.51–4.40) (9).

Comorbid depression in AD patients is associated with an increased risk of suicide attempts, relapse, treatment dropouts, and life dissatisfaction (10–12). For instance, earlier studies reported that compared with subjects with depression only, AD patients with depression had a suicide-specific standardized mortality ratio that was approximately two times higher, as well as more frequent episodes and more severe depression symptomatology (13–15). A more recent study showed that the co-occurrence of AD and depression negatively impacted quality of life and worsened physical functioning (16). Furthermore, AD aggravated the symptoms and prolonged the course of depression (17, 18). Two longitudinal studies revealed that problematic alcohol use increased the likelihood of persistent depression (19), and a reduction in drinking decreased the risk of depression (20). In turn, depressive symptoms heightened problem drinking and the risk of a relapse of AD (21). In summary, AD poses a potent risk for the development of depression and vice versa (22–24).

However, previous studies regarding comorbid depressive symptoms in AD patients were conducted in Western or African countries (6, 7, 25). To date, no published data on the prevalence and clinical correlates of comorbid depressive symptoms in AD patients in the Chinese Han population are available. Therefore, we conducted the present study to investigate the prevalence of and factors associated with depressive symptoms in AD patients in China.

## Materials and Methods

### Subjects

This study was conducted in eight hospitals with the psychiatric department in China. These eight hospitals are as follows: the Second Xiangya Hospital of Central South University, Henan Mental Hospital, Peking University Sixth Hospital, Shanghai Mental Health Center, West China Hospital of Sichuan University, Nanjing Brain Hospital, Hubei General Hospital, and the First Affiliated Hospital of Xian Jiaotong University. They are located in northern, northwestern, southwestern, central-southern, and eastern China. Therefore, our sample can represent Chinese hospitals to some extent. Patients were recruited from consecutive admissions to the psychiatric department of these eight hospitals in China. The inclusion criteria for the study were that the patients aged≥18 years, and the patients had the diagnosis of AD which was made by trained psychiatrists according to the DSM-IV criterion for alcohol dependence. A total of 413 patients met the inclusion criteria. However, 35 patients were excluded because they refused to participate in the whole study and could not complete the whole interview. Finally, 378 patients were enrolled and interviewed. These patients completed our self-developed questionnaire for demographic characteristics and drinking patterns, the BDI for depressive symptoms and the AUDIT for the severity of drinking.

The Institutional Review Board at the Second Xiangya Hospital of Central South University approved this study and all patients gave informed consent for participating in the study.

### Instruments

We used a self-developed questionnaire to assess 378 patients for demographic characteristics (gender, age, years of education, marital status, employment status, and monthly family income), and drinking patterns (age at first drink, daily consumption of pure alcohol, types of alcoholic beverages, and frequency of drinking).The Beck Depression Inventory (BDI) is a 21-item inventory that measures symptoms of depression. Based on the BDI total score, patients were classified into two groups: ≤13 = not depressed and ≥14 = depressed. Mild depression was defined as a total score of 14–19. Moderate depression was defined as a total score of 20–28, and severe depression was defined as a total score of 29 or above on the BDI (26). Suicidal ideation was measured using item I about suicide of the BDI (I don't have any thoughts of killing myself; I have thoughts of killing myself, but I would not carry them out; I would like to kill myself; I would kill myself if I had the chance). The Alcohol Use Disorders Identification Test (AUDIT) is a screening tool with 10 items about recent alcohol use, alcohol dependence symptoms, and alcohol-related problems. The maximum score of the AUDIT is 40 points. The AUDIT consists of two factors: a consumption factor (items 1–3) and an adverse consequence of drinking factor (items 4–10) (27, 28). The AUDIT has been standardized and validated for the Chinese population (29). The 10-item AUDIT-Chinese version was used in this study. Both the BDI and AUDIT were administered by trained and experienced psychiatrists. The diagnosis of AD was made for each participant by two trained psychiatrists according to the DSM-IV criterion for alcohol dependence, which requires three or more dependence items in twelve months (30).

### Statistical Analysis

All data were analyzed by SPSS version 20.0 for Windows. Testing for normality was accomplished by Kolmogorov-Smirnov one-sample test. Continuous data were presented as the mean ± standard deviation. The daily consumption data of pure alcohol were described as the median and interquartile range because these data were non-normally distributed. Categorical data were shown as frequencies and percentages. Differences in continuous variables were compared using Student's *t-*test, and categorical variables were compared using the chi-squared test. Logistic regression analysis with backward Wald method was used to compare depression and non-depression among the AD patients after controlling for the related variables (gender, age, marital status, years of education, employment status, monthly family income, age at first drink, and AUDIT total score). The significance level was set at *P* < 0.05.

## Results

### The Prevalence of Comorbid Depressive Symptoms in AD Patients

A total of 185 (48.9%, 95% CI: 43.9–54.0%) of 378 AD patients met the criteria for depressive symptoms (BDI total score ≥14), and 193 (52.1%) did not. Eighty (43.2%) of the 185 patients met the criteria for mild depressive symptoms, and 105 (56.8%) of the 185 patients met the criteria for moderate or severe depressive symptoms. Importantly, 19.6% (74/378) of the sample reported the presence of suicidal ideation. A total of 37.3% (69/185) of patients with comorbid depressive symptoms had suicidal ideation; the rate was 2.6% in patients without depressive symptoms. The difference in suicidal ideation between these two groups was significant (χ^2^ = 72.379, *P* < 0.001) (**Table 1**).

**Table 1:** Demographic and clinical features between the alcohol dependence (AD) patients with and without depression.

Characteristics	Depression (*n*= 185)	Non-depression (*n*= 193)	*t*/χ^2^	*P*
Males/females	171/14	187/6	3.747	0.053
Age (years)	41.8 ± 9.9	44.1 ± 10.0	2.182	0.030
Education (years)	11.0 ± 3.5	11.0 ± 3.6	0.127	0.899
Marital status			10.233	0.001
Stable	137 (74.1%)	168 (87.0%)		
Unstable	48 (25.9%)	25 (13.0%)		
Employment status			0.508	0.476
Employed	134 (72.4%)	146 (75.6%)		
Unemployed	51 (27.6%)	47 (24.4%)		
Monthly family income(US$)			3.193	0.203
<$446	82 (44.6%)	102 (53.7%)		
$446~$744	77 (41.8%)	68 (35.8%)		
>$744	25 (13.6%)	20 (10.5%)		
Age at first drink (years)	18.6 ± 4.9	18.4 ± 4.7	0.395	0.693
BDI- suicidal ideation	69 (37.3%)	5 (2.6%)	72.379	<0.001
AUDIT score				
Consumption subscore	11.2 ± 1.4	11.0 ± 1.6	0.802	0.423
Adverse consequences subscore	18.1 ± 5.6	15.8 ± 6.0	3.713	<0.001
Total score	29.3 ± 6.2	26.9 ± 6.6	3.575	<0.001

### Demographic Characteristics and Drinking Patterns of AD Patients

The whole sample consisted of 358 (94.7%) men and 20 (5.3%) women (male: female ratio=17.9:1.0). The mean age was 43.0 ± 10.0 years (men: 43.2 ± 9.7; women: 38.4 ± 13.4; *t* = 2.140, *P*=0.033), and the mean years of education was 11.0 ± 3.6 years (men: 11.0 ± 3.6; women: 11.9 ± 2.7; *t* = 1.193, *P* = 0.234). A total of 80.7% of the patients had a stable marital status, and 74.1% were employed.

Two hundred sixty-five (70.1%) of the patients drank alcoholic beverages at least once a day, 59 (15.6%) consumed 4–6/week, and 32 (8.5%) consumed 2–3/week. The median (interquartile range) daily consumption of pure alcohol was 100.8 g (59.4–168.0 g). A total of 378 patients consumed 61.4% of their alcohol in the form of strong distilled spirits (50% ethanol or above), 25.4% in the form of weaker distilled spirits (32%–45% ethanol), 9.8% in the form of beer, 2.1% in the form of rice wine, and 1.4% in the form of “other” beverages.

Mean scores on the AUDIT were as follows: consumption subscore, 11.1 ± 1.5; adverse consequences subscore, 17.0 ± 5.9; and total AUDIT score, 28.1 ± 6.5.

### Factors Associated With Comorbid Depressive Symptoms in AD Patients

As presented in **Table 1**, compared with those patients without depressive symptoms, those with depressive symptoms were younger, had more unstable marital status, had higher scores for the adverse consequences factor and had higher AUDIT total score (all *P* < 0.05). After adjustments were made for related factors (gender, age, marital status, years of education, employment status, monthly family income, age at first drink, and AUDIT total score), further logistic regression analysis with backward Wald method indicated that unstable marital status (OR=2.20, 95% CI 1.21–3.99, Waldχ^2^ = 6.670, *P*=0.010) and AUDIT total score (OR=1.07, 95% CI 1.03–1.11, Waldχ^2^ = 12.207, *P* < 0.001) remained significantly associated with comorbid depressive symptoms (**Table 2**).

**Table 2:** Risk factors generated by logistic regression analysis with depression as dependent variable.

	*B*	*SE*	Waldχ^2^	*P*	*OR*	95% CI
Marital status	0.786	0.304	6.670	0.010	2.20	1.21-3.99
AUDIT total score	0.068	0.019	12.207	<0.001	1.07	1.03-1.11
(Constant)	-0.601	1.165	0.266	0.606	–	–

## Discussion

To the best of our knowledge, the present study is the first national hospital-based survey to investigate the prevalence and demographic and clinical correlates of comorbid depressive symptoms in patients with AD in a Chinese Han population. We found that 48.9% of AD patients were depressed and that 56.8% of those depressed patients were moderate or severe depressed, suggesting a highly prevalent and serious depressive symptoms among AD patients. We also observed that the significant risk factors for depression in this population were unstable marital status and the AUDIT total score.

Our finding that the co-occurrence prevalence of depressive symptoms was 48.9% in AD patients closely corresponds with the findings of hospital-based studies conducted in other countries. For example, Ugochukwu et al. reported that the prevalence of major depression among 470 patients with alcohol use disorder was 45.8% in a tertiary hospital in Nigeria (6). Another study conducted by Odlaug and colleagues showed that the rate of depression among 2,979 AD patients was 43.1% in eight European countries (7). This consistency of results across multiple countries indicates a stable and high comorbidity of AD and depression. However, one recent survey employing the BDI revealed a much lower prevalence (24.7%) of depressive symptoms, with a cut-off of 21 points in Serbian patients with harmful alcohol use (31). This variation may be attributed to the inconsistent definitions of depression and alcohol use.

Importantly, many studies conducted in individuals from community populations also reported the comorbidity rate between AD and depression. For instance, according to the National Comorbidity Survey Replication, the prevalence of the co-occurrence of AD and major depression was 21.0% (1). In the Australian National Survey of Mental Health and Well Being, Burns and Teesson discovered that the prevalence of depression among residents with AD was 28% (5). One study conducted by Caetano in Puerto Rico reported that among those with alcohol use disorder, the rate of major depression was 23% (25). Another survey showed that the prevalence of depression in Nigerian college students with AD was 23.8% (4). In a prospective study, Briere et al. reported that the rate of cumulative alcohol use disorder and major depression comorbidity was 21% (12). The prevalence of depressive symptoms in AD patients was significantly higher than that in the general population, which might be explained by the greater severity of AD in hospital-based populations than in community-based populations. AD patients in psychiatric settings have more severe dependence symptoms, more life dissatisfaction, fewer family bonds, and worse social functioning and support than do community dwelling individuals (16). Therefore, the likelihood of AD patients developing depression is much higher than that of the general population. Further research conducted in both hospitals and communities at the same time is needed.

Recent evidence suggests that AD is associated with a significant risk of developing depression. According to Grant et al., the association between AD and depression was strong (OR=4.24, 95% CI 3.51–5.13) (3). Grant and colleagues updated this result in the National Epidemiologic Survey on Alcohol and Related Conditions, and found that the DSM-5 based alcohol use disorder was significantly correlated with major depression (OR=1.3, 95% CI 1.15–1.39) (32). Moreover, one study performed by Bazargan-Hejazi in a hospital in Los Angeles showed that alcohol abuse was associated with depressive symptoms, with an OR of 2.58(95% CI 1.51–4.40) (9). Furthermore, a meta-analysis of 22 studies conducted in non-clinical populations found that the pooled odds ratio for AD and major depression was 3.09 (95% CI 2.38–4.03) (8). Boden reviewed the literature and revealed that the presence of alcohol use disorder doubled the risk of major depression (33). Although the phenomenon of AD and depression comorbidity has been widely reported, the potential mechanisms underlying their association remain a controversial topic. Several possible explanations might account for the comorbidity between AD and depression. First, AD may precipitate the depressive symptoms caused by the pharmacological effects of alcohol. Moreover, AD patients may experience depressive symptoms as a result of a prolonged period of abstinence (31). Second, depression may occur as an emotional reaction to the adverse consequences of AD, such as unemployment (33–35). Third, individuals with depression drink alcohol for self-medication to alleviate depressive symptoms and eventually develop AD (36, 37). Finally, AD and depressive symptoms may occur simultaneously or develop independently due to shared biopsychosocial or genetic predisposition factors, such as poverty, genetic predisposition, or neuroimmune mechanisms (34). Many studies (38–42) found genetic overlap between AD and depression, suggesting that shared genetic liability possibly explains their comorbidity. For instance, Muench reported that the risk variant rs10514299 of major depressive disorder predicts the reward process of AD (39). Moreover, according to the survey from Zhou et al., SEMA3A variation was correlated with comorbid AD and major depression (41). Furthermore, studies have also found a neuroimmune interface in AD and depression comorbidity (43, 44). The mechanisms of comorbidity between AD and depression merit further investigation with a large sample size.

Previous studies (9, 25, 45–47) have revealed female gender, younger age, divorced or single status, unemployment, age at first drink, and severity of drinking problems to be independent predictors of comorbid depressive symptoms among patients with AD. In our study, gender, age, educational attainment, employment status, monthly family income, and age at first drink were not significantly associated with comorbid depression in logistic regression analysis. The potential reasons might be due to the relatively small sample size or recall bias, which need to be further studied. Consistent with other research, we found that unstable marital status and AUDIT total score were risk factors for the comorbidity of AD and depressive symptoms. For instance, according to the survey from Caetano et al., the severity of alcohol use disorder was positively correlated with the likelihood of major depression, and the AUDIT total score indicates the severity of drinking problems (25). Regarding marital status, studies report that marriage disruption was a powerful risk factor for major depression (48). Moreover, Hasin et al. observed that being widowed, separated, and divorced increased the risk of depression (46). The present study indicates an urgent need to provide psychotherapy to AD patients who are unstable in marriage or drinking too much to prevent the development of depression.

The present study found that gender had no significant relationship with AD patients with and without depression due to the relatively small sample size of female patients. Earlier studies showed that male gender is a protective factor against depression (25). One survey in patients with AD indicated that female patients showed more depressive symptoms than male patients did (45). Moreover, Cornelius et al. reported that the proportion of women was greater among depressed patients with AD than among non-depressed patients with AD (49). Another study discovered that among individuals with AD, women reported more depression than men did (50). The meta-analysis conducted by Conner concluded that among individuals with alcohol use disorder, women experienced more severe depressive symptoms than men did (47). In the general population, the rate of depression was higher among women than that among men (46, 51). We will enlarge the sample size to better illustrate the gender difference between AD patients with and without depression in the future.

We found that 19.6% of AD patients had suicidal ideation, and the rate (37.3%) was much higher in patients with comorbid depressive symptoms. Our result was consistent with previous studies. For example, Ziolkowski et al. reported that 12% of 510 patients with AD had suicidal ideation, and comorbidity with depressive symptoms increased the likelihood of suicidal thoughts (52). According to the study from Cornelius et al., depressed alcoholics had a much higher risk of suicidality than non-depressed alcoholics (49). Furthermore, one survey revealed that more than half of the patients with alcohol use disorder had engaged in at least one suicide attempt (53). Hence, clinicians should pay particular attention to AD patients with depression and offer a variety of interventions to prevent potential suicidal behaviors.

We observed that AD patients were more common among males than females, and the male/female ratio of AD patients (17.9) was especially striking. Our findings corresponded to those of earlier studies (32, 54, 55). For example, Hasin et al. reported that AD was significantly more prevalent in male adults (55). Furthermore, men consumed more alcohol and were more likely than women to drink harmfully (56, 57). Interestingly, we also observed that female AD patients were younger than male AD patients. Many studies have confirmed that although women had later onset of drinking, they developed AD more rapidly than men (58–60). The possible explanations for such gender differences in AD patients are biological factors, such as different alcohol pharmacokinetics and their effects on the brain and different levels of sex hormones (57). Moreover, sociocultural factors might also play an important role, including different tolerances of social drinking, socioeconomic status and social networks (61, 62).

The strength of our study is that it is multi-centered and was conducted in eight hospitals in China. Therefore, it could represent hospitals at different levels from diverse parts of China. This study also has several limitations. First, although the DSM-IV criterion for alcohol dependence was used, the diagnosis of major depressive disorder was not established. Depressive symptoms were identified only based on the BDI. Second, this study was cross-sectional. Therefore, the potential causality and direction between AD and depression could not be ascertained. Longitudinal studies are needed to elucidate this relationship. Third, some of our data were abstained by self-report. Hence, recall bias was unavoidable. Fourth, information about clinical factors, such as psychiatric comorbidity, duration of treatment and history of hospitalization, were not collected. Further research on these clinical factors and their association with depressive symptoms will be needed.

In conclusion, comorbid depressive symptoms are quite common among AD patients in China. Moreover, depression is associated with unstable marital status and the AUDIT total score. Considering the potential adverse consequences, particularly the high rate of suicidal ideation in patients with depressive symptoms, more attention needs to be devoted to the early detection and intervention of comorbid depressive symptoms in patients with AD.

## Data Availability Statement

The raw data supporting the conclusions of this article will be made available by the authors, without undue reservation.

## Ethics Statement

The studies involving human participants were reviewed and approved by The Institutional Review Board at the Second Xiangya Hospital of Central South University. The patients/participants provided their written informed consent to participate in this study.

## Author Contributions

WH designed the study. HH, ZZ, HC, KN, RuZ, WS, BL, HJ, WW, JD, MZ, ZY, JL, RoZ, SL, SX, XW, WF, and CG collected the sample and performed the literature review. HH and ZZ conducted the analyses and wrote the initial version of this manuscript. All authors edited, read, and approved the last version of this manuscript.

## Funding

This study was supported by the National Natural Science Foundation of China (Grant No.81901351), the Natural Science Foundation of Hubei Province of China (Grant No. 2019CFB269), the Health Commission of Hubei Province scientific research project (Grant No. WJ2019H352), and the Wuhan Municipal Health Commission scientific research project (Grant No. WX19Q05).

## Conflict of Interest

The authors declare that the research was conducted in the absence of any commercial or financial relationships that could be construed as a potential conflict of interest.

## References

[1] Pettinati HM O'Brien CP Dundon WD Current status of co-occurring mood and substance use disorders: a new therapeutic target. Am J Psychiatry (2013) 170:23–30. 10.1176/appi.ajp.2012.12010112 23223834 PMC3595612

[2] GBD 2017 DALYs and HALE Collaborators Global, regional, and national disability-adjusted life-years (DALYs) for 359 diseases and injuries and healthy life expectancy (HALE) for 195 countries and territories, 1990-2017: a systematic analysis for the Global Burden of Disease Study 2017. Lancet (2018) 392:1859–922. 10.1016/S0140-6736(18)32335-3 PMC6252083 30415748

[3] Grant BF Harford TC Comorbidity between DSM-IV alcohol use disorders and major depression: results of a national survey. Drug Alcohol Depend (1995) 39:197–206. 10.1016/0376-8716(95)01160-4 8556968

[4] Adewuya AO Prevalence of major depressive disorder in Nigerian college students with alcohol-related problems. Gen Hosp Psychiatry (2006) 28:169–73. 10.1016/j.genhosppsych.2005.09.002 16516068

[5] Burns L Teesson M Alcohol use disorders comorbid with anxiety, depression and drug use disorders. Findings from the Australian National Survey of Mental Health and Well Being. Drug Alcohol Depend (2002) 68:299–307. 10.1016/s0376-8716(02)00220-x 12393224

[6] Ugochukwu OC Donald CC Chukwuemeka SP Comorbidity of alcohol use disorder and depression among patients attending a tertiary hospital in the Niger Delta region of Nigeria. Neuroscience (2016) 4:38–42. 10.11648/j.ajpn.20160403.11

[7] Odlaug BL Gual A DeCourcy J Perry R Pike J Heron L Alcohol Dependence, Co-occurring Conditions and Attributable Burden. Alcohol Alcohol (2016) 51:201–9. 10.1093/alcalc/agv088 PMC4755551 26246514

[8] Lai HM Cleary M Sitharthan T Hunt GE Prevalence of comorbid substance use, anxiety and mood disorders in epidemiological surveys, 1990-2014: A systematic review and meta-analysis. Drug Alcohol Depend (2015) 154:1–13. 10.1016/j.drugalcdep.2015.05.031 26072219

[9] Bazargan-Hejazi S Bazargan M Gaines T Jemanez M Alcohol misuse and report of recent depressive symptoms among ED patients. Am J Emerg Med (2008) 26:537–44. 10.1016/j.ajem.2007.08.019 PMC5159686 18534281

[10] Hawton K Casanas ICC Haw C Saunders K Risk factors for suicide in individuals with depression: a systematic review. J Affect Disord (2013) 147:17–28. 10.1016/j.jad.2013.01.004 23411024

[11] Suter M Strik W Moggi F Depressive symptoms as a predictor of alcohol relapse after residential treatment programs for alcohol use disorder. J Subst Abuse Treat (2011) 41:225–32. 10.1016/j.jsat.2011.03.005 21546204

[12] Briere FN Rohde P Seeley JR Klein D Lewinsohn PM Comorbidity between major depression and alcohol use disorder from adolescence to adulthood. Compr Psychiatry (2014) 55:526–33. 10.1016/j.comppsych.2013.10.007 PMC4131538 24246605

[13] Park S Rim SJ Jo M Lee MG Kim CE Comorbidity of Alcohol Use and Other Psychiatric Disorders and Suicide Mortality: Data from the South Korean National Health Insurance Cohort, 2002 to 2013. Alcohol Clin Exp Res (2019) 43:842–49. 10.1111/acer.13989 30779437

[14] Carton L Pignon B Baguet A Benradia I Roelandt JL Vaiva G Influence of comorbid alcohol use disorders on the clinical patterns of major depressive disorder: A general population-based study. Drug Alcohol Depend (2018) 187:40–7. 10.1016/j.drugalcdep.2018.02.009 29626745

[15] Yoon G Petrakis IL Rosenheck RA Correlates of major depressive disorder with and without comorbid alcohol use disorder nationally in the veterans health administration. Am J Addict (2015) 24:419–26. 10.1111/ajad.12219 25950244

[16] Hassan AN Patients With Alcohol Use Disorder Co-Occurring With Depression and Anxiety Symptoms: Diagnostic and Treatment Initiation Recommendations. J Clin Psychiatry (2018) 79:69–71. 10.4088/JCP.17ac11999 29244266

[17] Awaworyi CS Farrell L Alcohol and depression: Evidence from the 2014 health survey for England. Drug Alcohol Depend (2017) 180:86–92. 10.1016/j.drugalcdep.2017.08.006 28886396

[18] Lazareck S Robinson JA Crum RM Mojtabai R Sareen J Bolton JM A longitudinal investigation of the role of self-medication in the development of comorbid mood and drug use disorders: findings from the National Epidemiologic Survey on Alcohol and Related Conditions (NESARC). J Clin Psychiatry (2012) 73:e588–93. 10.4088/JCP.11m07345 PMC4151244 22697205

[19] Carvalho AF Stubbs B Maes M Solmi M Vancampfort D Kurdyak PA Different patterns of alcohol consumption and the incidence and persistence of depressive and anxiety symptoms among older adults in Ireland: A prospective community-based study. J Affect Disord (2018) 238:651–8. 10.1016/j.jad.2018.06.041 29957483

[20] Knox J Scodes J Wall M Witkiewitz K Kranzler HR Falk D Reduction in non-abstinent WHO drinking risk levels and depression/anxiety disorders: 3-year follow-up results in the US general population. Drug Alcohol Depend (2019) 197:228–35. 10.1016/j.drugalcdep.2019.01.009 PMC6440807 30852375

[21] Chi FW Satre DD Weisner C Chemical dependency patients with cooccurring psychiatric diagnoses: service patterns and 1-year outcomes. Alcohol Clin Exp Res (2006) 30:851–9. 10.1111/j.1530-0277.2006.00100.x 16634854

[22] Regier DA Farmer ME Rae DS Locke BZ Keith SJ Judd LL Comorbidity of mental disorders with alcohol and other drug abuse. Results from the Epidemiologic Catchment Area (ECA) Study. JAMA (1990) 264:2511–8. 10.1001/jama.264.19.2511 2232018

[23] Swendsen JD Merikangas KR The comorbidity of depression and substance use disorders. Clin Psychol Rev (2000) 20:173–89. 10.1016/s0272-7358(99)00026-4 10721496

[24] Gilman SE Abraham HD A longitudinal study of the order of onset of alcohol dependence and major depression. Drug Alcohol Depen (2001) 63:277–86. 10.1016/S0376-8716(00)00216-7 11418232

[25] Caetano R Vaeth P Canino G Comorbidity of Lifetime Alcohol Use Disorder and Major Depressive Disorder in San Juan, Puerto Rico. J Stud Alcohol Drugs (2019) 80:546–51. 10.15288/jsad.2019.80.546 PMC6811725 31603756

[26] Beck AT Ward CH Mendelson M Mock J Erbaugh J An inventory for measuring depression. Arch Gen Psychiatry (1961) 4:561–71. 10.1001/archpsyc.1961.01710120031004 13688369

[27] Babor TF Higgins-Biddle JC Saunders JB Monteiro MG The Alcohol Use Disorders Identification Test. World Health Org Geneva (2001).

[28] Reinert DF Allen JP The alcohol use disorders identification test: an update of research findings. Alcohol Clin Exp Res (2007) 31:185–99. 10.1111/j.1530-0277.2006.00295.x 17250609

[29] Li Q Babor TF Hao W Chen X The Chinese translations of Alcohol Use Disorders Identification Test (AUDIT) in China: a systematic review. Alcohol Alcohol (2011) 46:416–23. 10.1093/alcalc/agr012 PMC3119458 21467046

[30] American Psychiatric Association Diagnostic and Statistical Manual of Mental Disorders. 4th ed. Washington, DC: American Psychiatric Association (1994).

[31] Pavkovic B Zaric M Markovic M Klacar M Huljic A Caricic A Double screening for dual disorder, alcoholism and depression. Psychiatry Res (2018) 270:483–9. 10.1016/j.psychres.2018.10.013 30326431

[32] Grant BF Goldstein RB Saha TD Chou SP Jung J Zhang H Epidemiology of DSM-5 Alcohol Use Disorder: Results From the National Epidemiologic Survey on Alcohol and Related Conditions III. JAMA Psychiat (2015) 72:757–66. 10.1001/jamapsychiatry.2015.0584 PMC5240584 26039070

[33] Boden JM Fergusson DM Alcohol and depression. Addiction. (2011) 106:906–14. 10.1111/j.1360-0443.2010.03351.x 21382111

[34] Cerda M Sagdeo A Galea S Comorbid forms of psychopathology: key patterns and future research directions. Epidemiol Rev (2008) 30:155–77. 10.1093/epirev/mxn003 18621743

[35] Hall W Degenhardt L Teesson M Reprint of “Understanding comorbidity between substance use, anxiety and affective disorders: Broadening the research base”. Addict Behav (2009) 34:795–9. 10.1016/j.addbeh.2009.03.040 19604645

[36] Hasin DS Endicott J Keller MB Alcohol problems in psychiatric patients: 5-year course. Compr Psychiatry (1991) 32:303–16. 10.1016/0010-440x(91)90078-q 1935019

[37] Schuckit MA Tipp JE Bergman M Reich W Hesselbrock VM Smith TL Comparison of induced and independent major depressive disorders in 2,945 alcoholics. Am J Psychiatry (1997) 154:948–57. 10.1176/ajp.154.7.948 9210745

[38] Foo JC Streit F Treutlein J Ripke S Witt SH Strohmaier J Shared genetic etiology between alcohol dependence and major depressive disorder. Psychiatr Genet (2018) 28:66–70. 10.1097/YPG.0000000000000201 29901528 PMC6039372

[39] Muench C Schwandt M Jung J Cortes CR Momenan R Lohoff FW The major depressive disorder GWAS-supported variant rs10514299 in TMEM161B-MEF2C predicts putamen activation during reward processing in alcohol dependence. Transl Psychiatry (2018) 8:131. 10.1038/s41398-018-0184-9 30006604 PMC6045574

[40] Torvik FA Rosenstrom TH Ystrom E Tambs K Roysamb E Czajkowski N Stability and change in etiological factors for alcohol use disorder and major depression. J Abnorm Psychol (2017) 126:812–22. 10.1037/abn0000280 PMC5546937 28541064

[41] Zhou H Polimanti R Yang BZ Wang Q Han S Sherva R Genetic Risk Variants Associated With Comorbid Alcohol Dependence and Major Depression. JAMA Psychiat (2017) 74:1234–41. 10.1001/jamapsychiatry.2017.3275 PMC6331050 29071344

[42] Polimanti R Peterson RE Ong JS MacGregor S Edwards AC Clarke TK Evidence of causal effect of major depression on alcohol dependence: findings from the psychiatric genomics consortium. Psychol Med (2019) 49:1218–26. 10.1017/S0033291719000667 PMC6565601 30929657

[43] Kelley KW Dantzer R Alcoholism and inflammation: neuroimmunology of behavioral and mood disorders. Brain Behav Immun (2011) 25 Suppl 1:S13–20. 10.1016/j.bbi.2010.12.013 PMC4068736 21193024

[44] Neupane SP Neuroimmune Interface in the Comorbidity between Alcohol Use Disorder and Major Depression. Front Immunol (2016) 7:655. 10.3389/fimmu.2016.00655 28082989 PMC5186784

[45] Palma-Alvarez RF Rodriguez-Cintas L Abad AC Sorribes M Ros-Cucurull E Robles-Martinez M Mood Disorders and Severity of Addiction in Alcohol-Dependent Patients Could Be Mediated by Sex Differences. Front Psychiatry (2019) 10:343. 10.3389/fpsyt.2019.00343 31214056 PMC6554686

[46] Hasin DS Goodwin RD Stinson FS Grant BF Epidemiology of major depressive disorder: results from the National Epidemiologic Survey on Alcoholism and Related Conditions. Arch Gen Psychiatry (2005) 62:1097–106. 10.1001/archpsyc.62.10.1097 16203955

[47] Conner KR Pinquart M Gamble SA Meta-analysis of depression and substance use among individuals with alcohol use disorders. J Subst Abuse Treat (2009) 37:127–37. 10.1016/j.jsat.2008.11.007 PMC4864601 19150207

[48] Kendler KS Hettema JM Butera F Gardner CO Prescott CA Life event dimensions of loss, humiliation, entrapment, and danger in the prediction of onsets of major depression and generalized anxiety. Arch Gen Psychiatry (2003) 60:789–96. 10.1001/archpsyc.60.8.789 12912762

[49] Cornelius JR Salloum IM Mezzich J Cornelius MD Fabrega HJ Ehler JG Disproportionate suicidality in patients with comorbid major depression and alcoholism. Am J Psychiatry (1995) 152:358–64. 10.1176/ajp.152.3.358 7864260

[50] King AC Bernardy NC Hauner K Stressful events, personality, and mood disturbance: gender differences in alcoholics and problem drinkers. Addict Behav (2003) 28:171–87. 10.1016/s0306-4603(01)00264-7 12507535

[51] Kessler RC Berglund P Demler O Jin R Koretz D Merikangas KR The epidemiology of major depressive disorder: results from the National Comorbidity Survey Replication (NCS-R). JAMA. (2003) 289:3095–105. 10.1001/jama.289.23.3095 12813115

[52] Ziolkowski M Czarnecki D Chodkiewicz J Gasior K Juczynski A Biedrzycka A Suicidal thoughts in persons treated for alcohol dependence: The role of selected demographic and clinical factors. Psychiatry Res (2017) 258:501–5. 10.1016/j.psychres.2017.08.089 28893411

[53] Morin J Wiktorsson S Marlow T Olesen PJ Skoog I Waern M Alcohol use disorder in elderly suicide attempters: a comparison study. Am J Geriatr Psychiatry (2013) 21:196–203. 10.1016/j.jagp.2012.10.020 23343493

[54] Teesson M Hall W Slade T Mills K Grove R Mewton L Prevalence and correlates of DSM-IV alcohol abuse and dependence in Australia: findings of the 2007 National Survey of Mental Health and Wellbeing. ADDICTION (2010) 105:2085–94. 10.1111/j.1360-0443.2010.03096.x 20707771

[55] Hasin DS Stinson FS Ogburn E Grant BF Prevalence, correlates, disability, and comorbidity of DSM-IV alcohol abuse and dependence in the United States: results from the National Epidemiologic Survey on Alcohol and Related Conditions. Arch Gen Psychiatry (2007) 64:830–42. 10.1001/archpsyc.64.7.830 17606817

[56] Wilsnack RW Wilsnack SC Kristjanson AF Vogeltanz-Holm ND Gmel G Gender and alcohol consumption: patterns from the multinational GENACIS project. Addiction. (2009) 104:1487–500. 10.1111/j.1360-0443.2009.02696.x PMC2844334 19686518

[57] Erol A Karpyak VM Sex and gender-related differences in alcohol use and its consequences: Contemporary knowledge and future research considerations. Drug Alcohol Depend (2015) 156:1–13. 10.1016/j.drugalcdep.2015.08.023 26371405

[58] Mann K Ackermann K Croissant B Mundle G Nakovics H Diehl A Neuroimaging of gender differences in alcohol dependence: are women more vulnerable? Alcoholism: Clin Exp Res (2005) 29:896–901. 10.1097/01.ALC.0000164376.69978.6B 15897736

[59] Ehlers CL Gizer IR Vieten C Gilder A Gilder DA Stouffer GM Age at regular drinking, clinical course, and heritability of alcohol dependence in the San Francisco family study: a gender analysis. Am J Addict (2010) 19:101–10. 10.1111/j.1521-0391.2009.00021.x PMC2835733 20163381

[60] Diehl A Croissant B Batra A Mundle G Nakovics H Mann K Alcoholism in women: is it different in onset and outcome compared to men? Eur Arch Psy Clin N (2007) 257:344–51. 10.1007/s00406-007-0737-z 17629733

[61] Im PK Millwood IY Guo Y Du H Chen Y Bian Z Patterns and trends of alcohol consumption in rural and urban areas of China: findings from the China Kadoorie Biobank. BMC Public Health (2019) 19:217. 10.1186/s12889-019-6502-1 30786877 PMC6383236

[62] Tang YL Xiang XJ Wang XY Cubells JF Babor TF Hao W Alcohol and alcohol-related harm in China: policy changes needed. Bull World Health Organ (2013) 91:270–6. 10.2471/BLT.12.107318 PMC3629448 23599550

